# *Notes from the Field:* *Salmonella* Oranienburg Infection Linked to Consumption of Rattlesnake Pills — Kansas and Texas, 2017

**DOI:** 10.15585/mmwr.mm6717a4

**Published:** 2018-05-04

**Authors:** Lyndsay Bottichio, Lindsey Martin Webb, Greg Leos, Beth Tolar, Natasha Dowell, Colin Basler

**Affiliations:** ^1^Division of Foodborne, Waterborne, and Environmental Diseases, National Center for Emerging and Zoonotic Diseases, CDC; ^2^Kansas Department of Health and Environment; ^3^Texas Department of State Health Services.

In November 2017, as part of a salmonellosis illness investigation, the Texas Department of State Health Services collected a bottle of rattlesnake pills from a patient’s home. The Texas Department of State Health Services then contacted CDC to report that a sample of these rattlesnake pills yielded *Salmonella* Oranienburg. The *Salmonella* serotype isolated from the patient was not related by pulsed-field gel electrophoresis (PFGE) to that isolated from the rattlesnake pills. PulseNet, the national molecular subtyping network for foodborne disease surveillance, identified numerous isolates with a PFGE pattern indistinguishable from that of the rattlesnake pill isolate.* Whole genome sequencing (WGS) indicated that the *Salmonella* found in the sample of rattlesnake pills was closely related genetically to an isolate from a patient in Kansas.^†^ This close genetic relationship makes it likely that the Kansas patient became ill from consumption of rattlesnake pills. Because the *Salmonella* serotype isolated from the patient was not related by PFGE to that isolated from the rattlesnake pills, the definitive cause of the Texas patient’s illness was not ascertained; this patient was unable to be reinterviewed, and no additional samples were able to be collected.

Rattlesnake pills, which contain encapsulated dehydrated and pulverized rattlesnake meat, are marketed as remedies for various conditions, ranging from cancer to acne. Rattlesnake pills are not approved by the Food and Drug Administration and are sometimes labeled by the manufacturer as “natural.” Rattlesnake pills can be found in alternative medicine and health food stores and roadside markets and can be purchased through Internet retailers.^§^

The Kansas patient was initially interviewed using a standard enteric illness questionnaire, which included a question about vitamins and supplements. This patient indicated taking other supplements but did not report taking rattlesnake pills. After learning the patient’s isolate was closely related to the rattlesnake pill isolate by WGS, the Kansas Department of Health and Environment reinterviewed the patient on December 17, 2017, specifically asking about less common supplements including rattlesnake pills. During the second interview, the patient reported having traveled to the State of Chihuahua, Mexico, and purchasing “pastillas de víbora de cascabel” (rattlesnake pills). The patient believed the pills to be homemade and consumed five pills; the patient had no remaining pills, so it could not be determined whether the source was the same as for the Texas patient’s pills. At the time of this report, no additional infections of *Salmonella* related to the Kansas patient or Texas pill sample have been identified; no other isolates were related to rattlesnake pills by WGS. 

Reptiles and their meat can carry *Salmonella* species that cause illness. Previous outbreak investigations have identified rattlesnake pills as a source of human *Salmonella* infections; a majority of illnesses occurred in persons with cancer (*1*–*4*) who were taking the rattlesnake pills for medicinal purposes, and most of those infections were associated with *S*. *arizonae*. This is the first report of *S*. Oranienburg infection associated with consumption of rattlesnake pills. Persons with compromised immune systems, including those with human immunodeficiency virus infection or who are receiving chemotherapy, pregnant women, children aged <5 years, and adults aged >60 years are more likely to develop a severe *Salmonella* infection that can result in hospitalization or even death from consuming a contaminated food or supplement.^¶^ The Food and Drug Administration does not review rattlesnake pills for safety or effectiveness.** Persons choosing to take rattlesnake pills, especially persons at higher risk for severe *Salmonella* infections, should be aware of the risk for salmonellosis associated with their consumption. Consultation with a licensed health care provider to discuss potential risks and benefits is recommended before taking any supplements.

## Footnotes

* https://www.cdc.gov/pulsenet.

† https://www.cdc.gov/pulsenet/pathogens/wgs.html.

§ https://www.fda.gov/ForConsumers/ConsumerUpdates/ucm466588.htm.

¶ https://www.cdc.gov/salmonella/general/index.html#two.

** https://www.fda.gov/Food/DietarySupplements/UsingDietarySupplements/ucm109760.htm.
